# 2,2′-[2,3,5,6-Tetra­methyl-*p*-phenyl­ene­bis(methyl­enethio)]bis­(pyridine *N*-oxide)

**DOI:** 10.1107/S1600536807068766

**Published:** 2008-01-09

**Authors:** B. Ravindran Durai Nayagam, Samuel Robinson Jebas, Selvarathi Grace, Dieter Schollmeyer

**Affiliations:** a Department of Chemistry, Popes College, Sawyerpuram, Tamilnadu, India; b Department of Physics, Karunya University, Coimbatore 641114, India; c Institut für Organische Chemie, Universität Mainz, Duesbergweg 10-14, 55099 Mainz, Germany

## Abstract

Mol­ecules of the title compound, C_22_H_24_N_2_O_2_S_2_, lie across centres of inversion. The two thio­pyridine *N*-oxide groups adopt a stepped *trans* configuration with respect to the benzene ring, by virtue of the symmetry. The oxopyridinium ring forms a dihedral angle of 79.9 (2)° with the benzene ring. The crystal structure is stabilized by a strong π–π inter­action between the pyridinium rings of adjacent mol­ecules [ring centroid–centroid distance = 3.464 (3) Å].

## Related literature

For bond-length data, see: Allen *et al.* (1987 ▶). For biological activities of *N*-oxide derivatives, see: Bovin *et al.* (1992 ▶); Katsuyuki *et al.* (1991 ▶); Leonard *et al.* (1955 ▶); Lobana & Bhatia (1989 ▶); Symons & West (1985 ▶). For a related structure, see: Hartung *et al.* (1996 ▶).
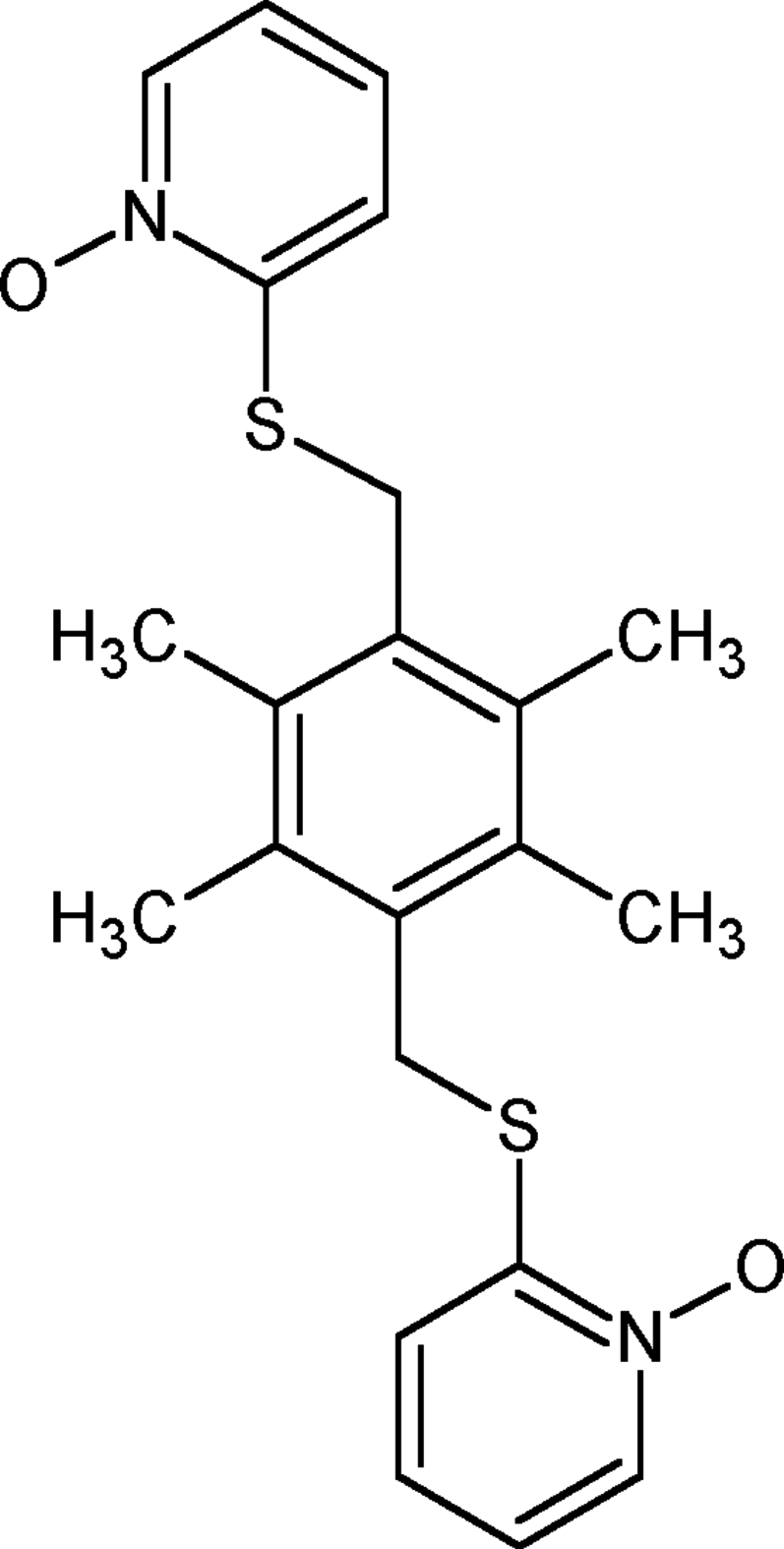


## Experimental

### 

#### Crystal data

C_22_H_24_N_2_O_2_S_2_
*M* *_r_* = 412.55
Monoclinic, 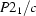

*a* = 11.8431 (13) Å
*b* = 9.0108 (9) Å
*c* = 9.7551 (10) Å
β = 112.611 (9)°
*V* = 961.01 (17) Å^3^
*Z* = 2
Cu *K*α radiation
μ = 2.68 mm^−1^
*T* = 193 (2) K
0.10 × 0.10 × 0.05 mm

#### Data collection

Enraf–Nonius CAD-4 diffractometer
Absorption correction: ψ scan (North *et al.*, 1968 ▶) *T*_min_ = 0.80, *T*_max_ = 0.87
1929 measured reflections
1813 independent reflections
1200 reflections with *I* > 2σ(*I*)
*R*_int_ = 0.077
3 standard reflections frequency: 60 min intensity decay: 3%

#### Refinement

*R*[*F* ^2^ > 2σ(*F* ^2^)] = 0.057
*wR*(*F* ^2^) = 0.170
*S* = 0.99
1813 reflections
129 parameters
H-atom parameters constrained
Δρ_max_ = 0.42 e Å^−3^
Δρ_min_ = −0.41 e Å^−3^

### 

Data collection: *CAD-4 EXPRESS* (Enraf–Nonius, 1994 ▶); cell refinement: *CAD-4 EXPRESS*; data reduction: *XCAD4* (Harms & Wocadlo, 1995 ▶); program(s) used to solve structure: *SHELXS97* (Sheldrick, 2008 ▶); program(s) used to refine structure: *SHELXL97* (Sheldrick, 2008 ▶); molecular graphics: *ORTEP-3 for Windows* (Farrugia, 1997 ▶); software used to prepare material for publication: *SHELXL97*.

## Supplementary Material

Crystal structure: contains datablocks global, I. DOI: 10.1107/S1600536807068766/ci2548sup1.cif

Structure factors: contains datablocks I. DOI: 10.1107/S1600536807068766/ci2548Isup2.hkl

Additional supplementary materials:  crystallographic information; 3D view; checkCIF report

## Figures and Tables

**Fig. 1.**
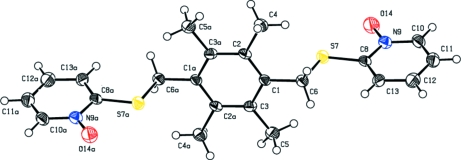
The molecular structure of the title compound, showing 50% probability displacement ellipsoids. Atoms labelled with the suffix a are generated by the symmetry operations (1 - x, 1 - y, 1 - z).

## supplementary crystallographic information

### Comment

N-Oxides and their derivatives show a broad spectrum of biological activity, such as antifungal, antibacterial, antimicrobial and antibiotic activities (Lobana & Bhatia, 1989; Symons *et al.*, 1985). These compounds are also found to be involved in DNA strand scission under physiological conditions (Katsuyuki *et al.*, 1991; Bovin *et al.*, 1992). Pyridine N-oxides bearing a sulfur group in position 2 display significant antimicrobial activity (Leonard *et al.*, 1955).

The asymmetric unit of the title compound consists of one half of a centrosymmetric molecule. The two thiopyridine-N-oxide groups adopt a stepped *trans* conformation with respect to the benzene ring, by virtue of the symmetry. The oxopyridinium ring forms a dihedral angle of 79.9 (2)° with the benzene ring. The N—O bond length is in good agreement with the mean value of 1.304 (15)Å reported in the literature for pyridine N-oxides (Allen *et al.*, 1987). As observed in a similar structure (Hartung *et al.*, 1996), the S atom is bent significantly towards the N-oxide O atom [N9—C8—S7 = 111.4 (3)°].

The crystal packing is stabilized by a strong π-π interaction between the pyridinium rings of adjacent molecules at (*x*, *y*, *z*) and (-*x*, 2 - *y*, -*z*), with a ring centroid to centroid distance of 3.464 (3) Å.

### Experimental

A mixture of 1,4-bis(bromomethyl)durene (0.320, 1 mmol) and 1-hydroxypyridine-2-thione sodium salt (0.298,2 mmol) in water (30 ml) and methanol (30 ml) was heated at 333 K with stirring for 30 min. The compound formed was filtered off, and dried (0.34 g, 82%). The compound was recrystallized from chloroform-methanol (1:2 *v*/*v*).

### Refinement

C-bound H atoms were placed in calculated positions [C—H = 0.95 Å (aromatic), 0.98 Å (methylene), and 0.99 Å (methyl)] and refined in the riding-model approximation, with *U*_iso_(H)=1.2–1.5*U*_eq_(C).

### Crystal data

**Table:**

C_22_H_24_N_2_O_2_S_2_	*F*_000_ = 436
*M**_r_* = 412.55	*D*_x_ = 1.426 Mg m^−^^3^
Monoclinic, *P*2_1_/*c*	Cu *K*α radiation λ = 1.54178 Å
Hall symbol: -P 2ybc	Cell parameters from 25 reflections
*a* = 11.8431 (13) Å	θ = 15–29.3º
*b* = 9.0108 (9) Å	µ = 2.68 mm^−^^1^
*c* = 9.7551 (10) Å	*T* = 193 (2) K
β = 112.611 (9)º	Block, colourless
*V* = 961.01 (17) Å^3^	0.10 × 0.10 × 0.05 mm
*Z* = 2	

### Data collection

**Table:**

Enraf–Nonius CAD-4 diffractometer	θ_max_ = 70º
ω/2θ scans	θ_min_ = 4.0º
Absorption correction: ψ scan(North *et al.*, 1968)	*h* = −14→13
*T*_min_ = 0.80, *T*_max_ = 0.87	*k* = 0→10
1929 measured reflections	*l* = 0→11
1813 independent reflections	3 standard reflections
1200 reflections with *I* > 2σ(*I*)	every 60 min
*R*_int_ = 0.077	intensity decay: 3%

### Refinement

**Table:**

Refinement on *F*^2^	H-atom parameters constrained
Least-squares matrix: full	*w* = 1/[σ^2^(*F*_o_^2^) + (0.1008*P*)^2^] where *P* = (*F*_o_^2^ + 2*F*_c_^2^)/3
*R*[*F*^2^ > 2σ(*F*^2^)] = 0.057	(Δ/σ)_max_ < 0.001
*wR*(*F*^2^) = 0.170	Δρ_max_ = 0.42 e Å^−^^3^
*S* = 0.99	Δρ_min_ = −0.41 e Å^−^^3^
1813 reflections	Extinction correction: none
129 parameters	

### Special details

**Table:**

Geometry. All e.s.d.'s (except the e.s.d. in the dihedral angle between two l.s. planes) are estimated using the full covariance matrix. The cell e.s.d.'s are taken into account individually in the estimation of e.s.d.'s in distances, angles and torsion angles; correlations between e.s.d.'s in cell parameters are only used when they are defined by crystal symmetry. An approximate (isotropic) treatment of cell e.s.d.'s is used for estimating e.s.d.'s involving l.s. planes.

### Fractional atomic coordinates and isotropic or equivalent isotropic displacement parameters (Å^2^)

**Table:**

	*x*	*y*	*z*	*U*_iso_*/*U*_eq_	
C1	0.4444 (3)	0.6186 (4)	0.4063 (4)	0.0220 (8)	
C2	0.5351 (3)	0.6459 (4)	0.5463 (4)	0.0234 (8)	
C3	0.4077 (3)	0.4715 (4)	0.3606 (4)	0.0243 (8)	
C4	0.5706 (4)	0.8042 (4)	0.5965 (4)	0.0305 (9)	
H4A	0.5049	0.8714	0.5374	0.046*	
H4B	0.646	0.8299	0.583	0.046*	
H4C	0.5838	0.8136	0.7017	0.046*	
C5	0.3044 (4)	0.4452 (4)	0.2124 (4)	0.0347 (10)	
H5A	0.2428	0.5233	0.1942	0.052*	
H5B	0.267	0.3484	0.2132	0.052*	
H5C	0.3365	0.447	0.1336	0.052*	
C6	0.3835 (3)	0.7458 (4)	0.3017 (4)	0.0258 (8)	
H6A	0.3528	0.7114	0.1973	0.031*	
H6B	0.4429	0.827	0.3138	0.031*	
S7	0.25697 (9)	0.81142 (11)	0.34802 (10)	0.0290 (3)	
C8	0.1962 (3)	0.9526 (4)	0.2167 (4)	0.0254 (8)	
N9	0.1042 (3)	1.0262 (4)	0.2392 (4)	0.0293 (7)	
C10	0.0488 (4)	1.1435 (4)	0.1524 (5)	0.0340 (10)	
H10	−0.0123	1.1969	0.1725	0.041*	
C11	0.0792 (4)	1.1863 (4)	0.0363 (5)	0.0347 (9)	
H11	0.0394	1.2683	−0.0242	0.042*	
C12	0.1688 (4)	1.1083 (5)	0.0083 (5)	0.0358 (10)	
H12	0.1889	1.1346	−0.0739	0.043*	
C13	0.2287 (4)	0.9925 (4)	0.0998 (4)	0.0287 (9)	
H13	0.2918	0.9405	0.0826	0.034*	
O14	0.0730 (3)	0.9825 (4)	0.3471 (3)	0.0443 (8)	

### Atomic displacement parameters (Å^2^)

**Table:**

	*U*^11^	*U*^22^	*U*^33^	*U*^12^	*U*^13^	*U*^23^
C1	0.032 (2)	0.0160 (17)	0.0218 (18)	0.0012 (15)	0.0144 (16)	0.0009 (14)
C2	0.0280 (19)	0.0172 (17)	0.0262 (19)	−0.0026 (14)	0.0117 (15)	−0.0053 (15)
C3	0.0294 (19)	0.0227 (19)	0.0217 (17)	−0.0043 (15)	0.0106 (15)	−0.0028 (15)
C4	0.038 (2)	0.0189 (19)	0.033 (2)	−0.0065 (17)	0.0124 (18)	−0.0023 (17)
C5	0.044 (2)	0.025 (2)	0.031 (2)	−0.0021 (19)	0.0101 (19)	−0.0028 (18)
C6	0.0303 (19)	0.0221 (17)	0.026 (2)	0.0011 (16)	0.0116 (16)	−0.0001 (16)
S7	0.0373 (5)	0.0247 (5)	0.0282 (5)	0.0039 (4)	0.0160 (4)	0.0041 (4)
C8	0.028 (2)	0.0183 (18)	0.0265 (19)	−0.0018 (15)	0.0065 (16)	−0.0063 (15)
N9	0.0308 (17)	0.0260 (17)	0.0300 (17)	0.0000 (14)	0.0102 (14)	−0.0034 (14)
C10	0.030 (2)	0.025 (2)	0.038 (2)	0.0035 (16)	0.0031 (18)	−0.0069 (18)
C11	0.035 (2)	0.0224 (19)	0.037 (2)	−0.0048 (18)	0.0027 (17)	−0.0011 (19)
C12	0.039 (2)	0.031 (2)	0.034 (2)	−0.0059 (18)	0.0102 (18)	0.0004 (18)
C13	0.035 (2)	0.0233 (19)	0.0249 (19)	−0.0011 (16)	0.0086 (17)	0.0008 (16)
O14	0.055 (2)	0.0477 (19)	0.0421 (18)	0.0125 (16)	0.0316 (16)	0.0064 (15)

### Geometric parameters (Å, °)

**Table:**

C1—C2	1.398 (5)	C11—C12	1.384 (6)
C1—C3	1.412 (5)	C12—C13	1.379 (6)
C1—C6	1.520 (5)	C4—H4A	0.98
C2—C3^i^	1.389 (5)	C4—H4B	0.98
C2—C4	1.514 (5)	C4—H4C	0.98
C3—C2^i^	1.389 (5)	C5—H5A	0.98
C3—C5	1.511 (5)	C5—H5B	0.98
C6—S7	1.822 (4)	C5—H5C	0.98
S7—C8	1.752 (4)	C6—H6A	0.99
C8—N9	1.364 (5)	C6—H6B	0.99
C8—C13	1.384 (5)	C10—H10	0.95
N9—O14	1.303 (4)	C11—H11	0.95
N9—C10	1.355 (5)	C12—H12	0.95
C10—C11	1.369 (6)	C13—H13	0.95
			
C2—C1—C3	120.1 (3)	C2—C4—H4C	109
C2—C1—C6	120.8 (3)	H4A—C4—H4B	109
C3—C1—C6	119.2 (3)	H4A—C4—H4C	109
C3^i^—C2—C1	120.2 (3)	H4B—C4—H4C	109
C3^i^—C2—C4	120.1 (3)	C3—C5—H5A	109
C1—C2—C4	119.7 (3)	C3—C5—H5B	110
C2^i^—C3—C1	119.7 (3)	C3—C5—H5C	109
C2^i^—C3—C5	121.2 (3)	H5A—C5—H5B	109
C1—C3—C5	119.1 (3)	H5A—C5—H5C	109
C1—C6—S7	107.5 (2)	H5B—C5—H5C	110
C8—S7—C6	101.51 (18)	S7—C6—H6A	110
N9—C8—C13	119.9 (4)	S7—C6—H6B	110
N9—C8—S7	111.4 (3)	C1—C6—H6A	110
C13—C8—S7	128.7 (3)	C1—C6—H6B	110
O14—N9—C10	121.4 (4)	H6A—C6—H6B	108
O14—N9—C8	118.4 (3)	N9—C10—H10	119
C10—N9—C8	120.2 (4)	C11—C10—H10	119
N9—C10—C11	121.2 (4)	C10—C11—H11	120
C10—C11—C12	119.1 (4)	C12—C11—H11	120
C13—C12—C11	119.9 (4)	C11—C12—H12	120
C12—C13—C8	119.6 (4)	C13—C12—H12	120
C2—C4—H4A	109	C8—C13—H13	120
C2—C4—H4B	109	C12—C13—H13	120
			
C3—C1—C2—C3^i^	2.0 (6)	C6—S7—C8—C13	5.8 (4)
C6—C1—C2—C3^i^	−178.3 (3)	C13—C8—N9—O14	177.6 (3)
C3—C1—C2—C4	−177.7 (3)	S7—C8—N9—O14	−1.6 (4)
C6—C1—C2—C4	1.9 (5)	C13—C8—N9—C10	−3.5 (5)
C2—C1—C3—C2^i^	−2.0 (6)	S7—C8—N9—C10	177.3 (3)
C6—C1—C3—C2^i^	178.3 (3)	O14—N9—C10—C11	−177.9 (4)
C2—C1—C3—C5	176.5 (3)	C8—N9—C10—C11	3.2 (6)
C6—C1—C3—C5	−3.1 (5)	N9—C10—C11—C12	−0.4 (6)
C2—C1—C6—S7	−86.0 (4)	C10—C11—C12—C13	−2.1 (6)
C3—C1—C6—S7	93.6 (4)	C11—C12—C13—C8	1.8 (6)
C1—C6—S7—C8	−178.8 (2)	N9—C8—C13—C12	1.0 (6)
C6—S7—C8—N9	−175.2 (3)	S7—C8—C13—C12	−180.0 (3)

Symmetry codes: (i) −*x*+1, −*y*+1, −*z*+1.
